# Deletion of *NRXN1α* impairs long-range and local connectivity in amygdala fear circuit

**DOI:** 10.1038/s41398-020-00926-y

**Published:** 2020-07-19

**Authors:** Douglas Asede, Asnel Joseph, McLean M. Bolton

**Affiliations:** Disorders of Neural Circuit Function, Max Planck Florida Institute for Neuroscience Jupiter, Jupiter, FL USA

**Keywords:** Autism spectrum disorders, Learning and memory

## Abstract

Neurexins are a family of presynaptic cell adhesion proteins that regulate synaptic structure and maintain normal synaptic transmission. Mutations in the α-isoform of neurexin1-gene (*NRXN1α*) are linked with cognitive and emotional dysregulation, which are heavily dependent on the amygdala and medial prefrontal cortex (mPFC). It is however not known whether deletion of *NRXN1α* gene affect specific synaptic elements within the amygdala microcircuit and connectivity with mPFC. In this study, we show that *NRXN1α* deletion impairs synaptic transmission between the dorsal medial prefrontal cortex (dmPFC) and basal amygdala (BA) principal neurons. Stimulation of dmPFC fibers resulted in reduced paired pulse ratio (PPR) and AMPA/NMDA ratio at dmPFC to BA synapses in *NRXN1α*-knockout (KO) (*NRXN1α* KO) mice suggestive of pre- and postsynaptic deficits but there was no change at the lateral amygdala (LA) to BA synapses following LA stimulation. However, feedforward inhibition from either pathway was significantly reduced, suggestive of input-independent deficit in GABAergic transmission within BA. We further analyzed BA inhibitory network and found reduced connectivity between BA GABAergic and glutamatergic neurons in *NRXN1α* KO mice. As this circuit is tightly linked with fear regulation, we subjected *NRXN1α* KO and WT mice to discriminative fear conditioning and found a deficit in fear memory retrieval in *NRXN1α* KO mice compared with WT mice. Together, we provide novel evidence that deletion of *NRNX1α* disrupts amygdala fear circuit.

## Introduction

Proper formation and maintenance of synaptic circuits is required for normal central nervous system function^1,2^. These circuits consist of intricate synaptic connections supported by cell adhesion molecules such as neurexins^3,4^. The mammalian genome contains three neurexin genes (*NRXN1*, *NRXN2*, and *NRXN3*), each of which has two independent promoters resulting in a large α (α-NRXN) and a small β-neurexin protein isoform (β-NRXN)^5,6^. Located mainly on presynaptic sites, neurexins interact with their postsynaptic partners, forming *trans*-synaptic complexes at excitatory and inhibitory synapses, to promote proper synapse specification, establishment, maturation, and plasticity^7–11^. Supporting this notion, triple knockout (KO) of *α-NRXNs* in mice resulted in an impairment in synaptic transmission and short-term plasticity in several brain regions, demonstrating their essential role at synapses^12^. Differential expression of members of the neurexin family among different classes of neurons and the resulting heterogeneity in *trans*-synaptic binding associations may contribute to synapse specificity and to the diversity of synaptic properties^13,14^. The presence of five alternative splice sites on *α-NRXNs*, two of which are also present on the *β-NRXNs*, adds rich combinatorial possibilities to synapse diversity^15^. This complexity increases the potential link between *α-NRXN* mutations and behavioral impairments.

Because of the prevalence of cognitive impairment and emotional dysregulation in disorders linked with *NRXN1α* mutations, related brain regions such as the medial prefrontal cortex (mPFC) and amygdala have been under the research spotlight^6,16–19^. To investigate amygdala-dependent behaviors such as emotional (fear) memories, pavlovian fear conditioning is widely used, during which an animal learns to associate a previously neutral conditioned stimulus (CS) with an aversive unconditioned stimulus (US). After several pairings, the CS acquires aversive properties and can subsequently be used to retrieve fear memories^20^. Acquisition of fear memories requires the convergence of synaptic inputs representing the CS and US onto glutamatergic neurons in the lateral amygdala (LA)^21^. For conditioned fear to be expressed, CS information is relayed to the central output nucleus of the amygdala (CEA) via glutamatergic inputs to basal amygdala (BA) neurons and medial intercalated cells, indirectly leading to heightened CEA output and high fear state^22–24^. The ability to distinguish between a harmless stimulus and an aversion predictor, CS, indicates the level of fear memory accuracy (discrimination)^25^. This and effective fear memory regulation require the reciprocal interaction between the BA and mPFC^25–29^. Evidence suggests that areas of mPFC play opposing role in fear; ventral and dorsal mPFC (dmPFC), which includes prelimbic region (PL), suppress and facilitate fear-related freezing, respectively^30,31^. The dmPFC fibers strongly innervate BA and elicit monosynaptic response upon stimulation, thereby promoting fear expression^31^. Although this synaptic network plays a critical role in regulating emotional response, it is not known whether specific synaptic elements and pathways within the fear circuit are disrupted by mutations in *NRXN* genes. As a synapse class-specific expressional diversity of neurexins makes them suitable candidates to differentially regulate these elements, we therefore ask whether mutations in a high-confidence risk gene such as *NRXN1α* could perturb local connections within the amygdala and/or long-range interactions with dmPFC. Using electrophysiological and behavioral techniques, we found input-specific deficits in excitatory transmission, global reduction in inhibitory transmission in BA, and impairment in fear memory retrieval in *NRXN1α* KO mice.

## Materials and methods

### Animals

*NRXN1α* heterozygote mice (+/−) in *C57BL/6J* genetic background JAX (021777) were crossed to generate wild-type (WT) (+/+) and homozygote KO (−/−) experimental groups. To unequivocally distinguish amygdala glutamatergic neurons from GABAergic neurons during electrophysiology, *NRXN1α* line was crossed with GAD67-GFP mice^32^ obtained from Riken (RBRC03674). Animals were group-housed with food and water ad libitum, under a 12 : 12 h light/dark cycle but were isolated a week before experiments for individual handling and to avoid the possibility of post-shock induced aggression among mice. Multiple cohorts were used for experiments and each cohort consists of WT and KO mice, tested in randomized order. Investigator was blinded to the animal genotype during the experiments but was not when assessing the outcome. Animals were 9–12 weeks old at the time of experiments. All housing and experimental procedures were conducted according to the Guide for the Care and Use of Laboratory Animals from National Institute of Health under the approval of the Institutional Care and Use Committee of Max Planck Florida Institute for Neuroscience.

### Behavioral experiments

#### Fear conditioning

Adult male mice (9–12 weeks) underwent a 10 min habituation session in the conditioning context (A), consisting of a square arena and stainless steel grid floor encased in a white sound-attenuated box (35.5 cm high, 63.5 cm wide, 76 cm deep; Med Associates NIR-022MD) cleaned with 70% ethanol. Following a 120 s exploration period on day 2, mice were subjected to discriminative fear conditioning with 10 CS+ – US pairings and 10 randomly interleaved CS- (30–140 s interstimulus interval, ISI). The CS+ was a 30 s tone (50 ms pips at 0.9 Hz, 12 kHz, and 90 dB) co-terminating with the US (1 s scrambled foot shock, 0.5 mA). In our hand, this shock intensity (0.5 mA) did not induce active defensive behaviors such as jumping and escape behavior in our mice. The CS− was an unpaired 30 s continuous tone (10 kHz and 90 dB).

#### Fear recall

Twenty four hours after fear conditioning (day 3), animals were tested in context B, which consists of a smooth white acrylic insert (ENV-005-GFCW) instead of the grid floor, and a translucent black plastic triangular tent (ENV-008-IRT), cleaned with 0.1% Alconox. Following a 125 s exploration period, fear memory was tested with the same CS+ and CS− used during acquisition but fewer (five CS+ and five CS−). These stimuli were randomized (30–140 s ISI), so that the exact time of presentation was different from the time of presentation during acquisition, to avoid stimulus onset prediction.

#### Fear measurement

Freezing was used as proxy for fear behavior during the 30 s of tone presentation. Freezing score was measured with an automated analysis software (video Freeze, SOF-843), which uses motion analysis algorithm to generate a motion index from the digital video stream. A motion index threshold of 18 and a minimum freeze duration of 30 frames (1 s) were used to determine freezing, as these have been reported to yield a high correlation and excellent linear fit between computer and human scores across a broad range of conditions^33^. Percent freezing was calculated as the amount of time the mouse spend motionless during tone presentation divided by the tone duration. As the freezing responses of the mice attain steady state during the last four CSs, mean freezing data for both acquisition and fear memory retrieval are presented as the average of the last 4 CSs (Fig. 5b, c). Supplementary Fig. S4 contains moving average charts of all CSs.

Differences in motion activities could serve as potential confounds when using freezing behavior as a proxy for fear measurement. To test whether there is a difference in motion activities between WT and *NRXN1α*, we analyzed average motion in WT and KO mice during a 10 min habituation phase using VideoFreeze motion analysis software^33^ (Med Associates).

### Viruses and stereotactic injections

For dmPFC terminal stimulation in the amygdala, 5- to 7-week-old male and female mice maintained under isoflurane anesthesia were stereotaxically injected with AAV (adeno-associated virus)-hSyn-hChR2(H134R)-YFP (PennVector Core, Philadelphia, PA) into the dmPFC (prelimbic and cingulate regions) at the following coordinates from the bregma (in mm): posterior 1.9, lateral ±0.3, ventral −2.1. Although, this AAV virus infect both glutamatergic and GABAergic neurons, to our knowledge, GABAergic neurons in mPFC do not project to amygdala.

For inhibitory connection mapping experiments, we used perisomata-targeted channelrhodopsin-2 (ChR2), with pAAV-hSyn-hChR2(H134R)-EYFP (Addgene plasmid 26973) backbone. The proximal restriction and clustering signal of the mouse Kv2.1 was generated by automated gene synthesis and amplified by PCR. The resulting product was inserted into the BsrGI site at the C terminus of the ChR2-EYFP fusion protein sequence. To better visualize cells for stimulation during mapping experiments, we generated a bicistronic AAV construct consisting of hChR2 followed immediately by the Kv2.1 targeting sequence, a P2A ribosomal skipping sequence, and a histone 2B-mRuby2 fusion protein^34^. Full-strength AAV1-hSyn-ChR2-Kv2.1-P2A-H2B-mRuby2 (5 × 10^12^ units/ml) was injected into BA (posterior 1.4, lateral ±3.3, and ventral 4.9 in mm from the bregma). As our virus construct could infect both glutamatergic and GABAergic neurons, we sought to minimize potential confounds by isolating inhibitory responses in the presence of excitation blockers (1 µM NBQX, 50 µM L-APV).

Acute slices were prepared for ex vivo recording 4–6 weeks post injection.

### Slice preparation and patch-clamp recordings

Coronal brain slices (320 µm) were prepared in ice-cold cutting solution containing (in mM): 124 choline chloride, 26 NaHCO_3_, 2.5 KCl, 3.3 MgCl_2_, 1.2 NaH_2_PO4, 1 glucose, and 0.5 CaCl_2_. After cutting, slices were allowed to recover for 30 min at 32 °C and stored at room temperature in artificial cerebrospinal fluid (ACSF) containing(in mM): 124 NaCl, 26 NaHCO_3_, 3 KCl, 1.25 NaH_2_PO_4_, 20 glucose, 1 MgCl_2_, 2 CaCl_2_, 5 sodium ascorbate, 3 sodium pyruvate, and 2 thiourea. All solutions were constantly oxygenated with 95%O_2_/5%CO_2_. Slices containing the amygdala were transferred to a submersion recording chamber, superfused with oxygenated ACSF at a speed of 1–2 ml/min. Whole-cell patch-clamp recordings were performed using pipettes pulled from borosilicate glass capillaries (BF150-110-10, Sutter Instrument, USA) with resistances of 4–7 MΩ. For recording postsynaptic currents, we used Cs-methanesulphonate based internal solution containing (mM): 135 Cs-methanesulphonate, 6 NaCl, 10 HEPES, 0.6 EGTA, 4 MgATP, and 0.3 NaGTP (290–295 mOsm, pH 7.2–7.3). In some experiments, 0.3–0.5% biocytin was added to the internal recording solution.

LA inputs were evoked by bipolar tungsten electrodes (Science Products) and dmPFC terminals activated with 470 nm light pulses (0.2–3 ms, 0.50–1.10 mW/mm^2^) from a light-emitting diode (X-cite XLED, Lumen Dynamics) through the ×20 magnification and 1.0 numerical aperture (NA) objective of an upright microscope (Axio Examiner D1; Zeiss). If stimulation of a specific pathway did not elicit monosynaptic response or elicited multi-peak responses, the pathway was excluded from the analysis. To investigate excitation/inhibition ratio at dmPFC- or LA–BA pathway, evoked excitatory postsynaptic currents (EPSCs) were isolated in voltage‐clamp mode at −70 mV, which is the chloride-reversal potential in our internal solution, and inhibitory postsynaptic currents (IPSCs) were recorded at 0 mV in the same neuron in drug-free ACSF. To selectively characterize excitatory transmission, pure excitatory components were isolated at the −70 mV, in the presence of 10 µM Bicuculline. Evaluation of synaptic inputs from dmPFC to BA and from LA to BA neurons were conducted in the same experiments. However, if stimulation of a specific pathway did not elicit monosynaptic response or elicited multi-peak responses, the pathway was excluded from the analysis. Pathways were stimulated in alternation, at 20 s ISI. Stimulation intensities were adjusted to obtain comparable AMPA (α-amino-3-hydroxy-5-methyl-4-isoxazolepropionic acid) EPSC amplitudes in WT and KO mice, except in input–output experiments. Data were acquired with a Multiclamp 700B amplifier, Digidata1440, and Clampex software. Signals were filtered at 2 kHz and digitized at 5 kHz. Data were analyzed with an automated software NeuroMatic (http://www.neuromatic.thinkrandom.com) and custom-written macros in IgorPro (Wavemetrics).

### Circuit mapping and connection analysis

To specifically activate selected subset of neurons expressing soma-tagged ChR2 in multiple neurons, targeted pattern illumination was performed using a digital micromirror device (Mosaic 2, Andor Technologies UK) mounted on Axio Examiner D1 microscope (Zeiss) connected to an X-LED1 light source.

Nuclear fluorescent tag (mRuby) was used to identify ChR2-expressing neurons and corresponding circular spots (12 µm diameter) were placed on individual cells. To illuminate individual spots with 470 nm blue light, Andor iQ 3.0 software (Andor Technologies, UK) and X-Cite XLED1 software (Lumen Dynamics) were used to control the micromirror array and XLED light source, respectively. IPSCs in response to stimulated spots were recorded at 0 mV in the presence of excitatory blockers (1 µM NBQX, 50 µM L-APV).

Bicuculline methobromide was purchased from Tocris Bioscience (Bristol, UK). All other chemicals and drugs were obtained from Sigma-Aldrich (Missouri, USA).

### Immunostaining and confocal imaging

#### Perisomatic puncta

WT and KO mice were perfused with 4% paraformaldehyde in phosphate-buffered saline and whole brains were fixed overnight at +4 °C. Brains were sectioned at 70 µm and slices containing amygdala were selected for immunostaining. Immunostainings were performed with standard procedures using the following combination of primary and secondary antibodies: Mouse anti-Kv2.1 (Neuromab) and Alexa 568-conjugated goat anti-mouse (Invitrogen); Guinea pig anti-vesicular GABA transporter (VGAT) (Synaptic Systems) and Alexa 647-conjugated goat anti-guinea pig (Invitrogen); and rabbit anti-cannabinoid receptor type 1 (CB1R) (Cayman) and Alexa 488-conjugated goat anti-rabbit (Invitrogen). All antibodies were used at 1 : 1000 dilution. Slices were imaged using a laser-scanning microscope (LSM 780; Carl Zeiss Germany) with a ×40 magnification and 1.0 NA oil and pinhole set at 1.2 µm for channels. The *z*-stack images (30 µm) were acquired at 0.6 µm optical sections and single midplane images were used for analysis. Perisomatic puncta quantification was performed using Puncta analyzer v2.0 in ImageJ (National Institute of Health) as previously described^35^.

### Statistics

Data are presented as mean ± SEM. All statistical analyses were performed using Graphpad Prism (GraphPad Software). Analysis of variance was performed where applicable and *p*-values were adjusted with post hoc Bonferroni correction for multiple comparison. Data were considered significant if *p* < 0.05. Significance levels are denoted as follows: *p < 0.05, **p < 0.01, ***p < 0.001.

## Results

### *NRXN1α* KO resulted in a deficit in excitatory transmission at dmPFC to BA synapses but no change at LA to BA synapses

**Fig. 1:**
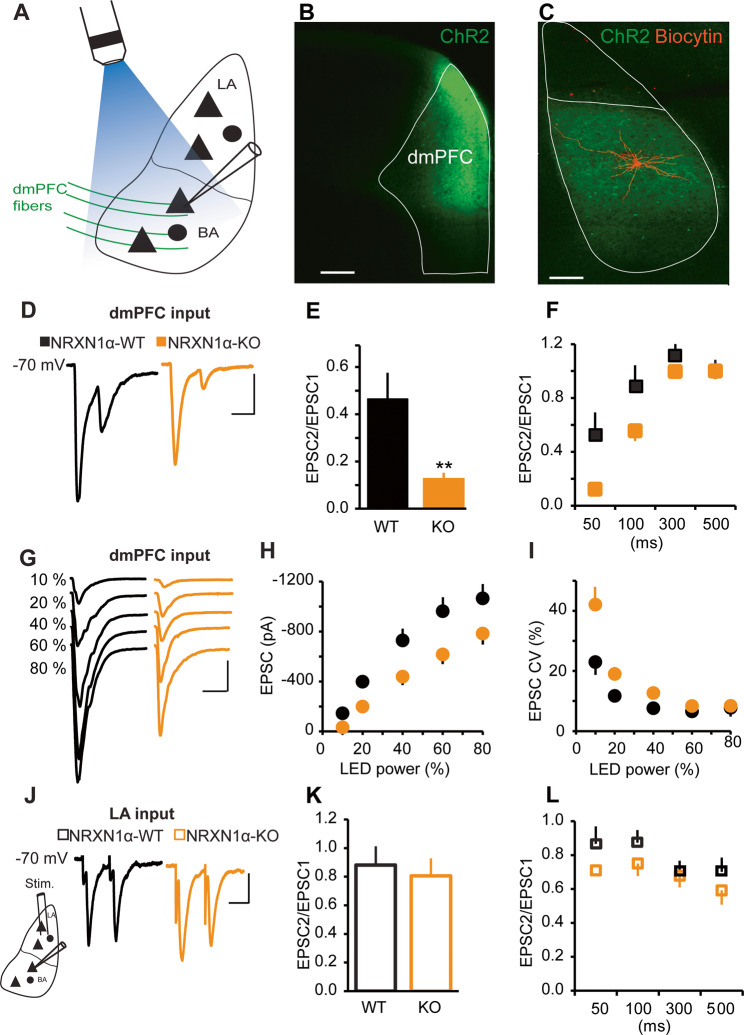
Input-specific alterations at dmPFC–BA synapse but not LA–BA synapse. **a**–**f** Properties of EPSCs evoked by light stimulation of dmPFC terminals in the BA. **a** Schematic diagram showing light activation of ChR2-expressing dmPFC fibers and whole-cell patch-clamp recording from a BA principal cell. **b** Virus injection site in dmPFC. Scale bar: 200 μm. **c** ChR-expressing dmPFC axons and a biocytin-labeled neuron in BA. Scale bar: 100 μm. **d** Sample traces of AMPA receptor mediated EPSC pairs recorded at −70 mV at 50 ms interstimulus interval (ISI). Scale bars: 30 ms, 40 pA. **e** Mean PPR at 50 ms ISI. Reduced PPR in KO mice. *n* = 14 cells in 4 mice for each group; **p < 0.005. **f** PPR at different ISIs. WT (*n* = 7 cells in 3 mice), KO (*n* = 10 cells in 3 mice); two-way ANOVA, F (1, 15) = 6.944, *p* < 0.05. **g**–**i** Decrease in input–output function at dmPFC–BA synapse in *NRXN1α*-KO mice. WT (*n* = 14 cells in 4 mice), KO (*n* = 13 cells in 3 mice). **g** Representative traces of AMPA EPSCs at −70 mV evoked by increasing LED power. Scale bars: 40 ms, 200pA. **h** AMPA current amplitude vs LED power. Decreased AMPA EPSC amplitude across stimulation intensities. Two-way ANOVA, F (1, 25) = 7.092, *p* < 0.05. **i** Concomitant increase in AMPA current CV in KO mice. Two-way ANOVA, F (1, 25) = 7.092, *p* < 0.001. **j**–**l** Synaptic responses of BA neurons to LA stimulation. **j** Sample traces of pairs of AMPA receptor mediated EPSCs at 50 ms ISI evoked by bipolar electrode stimulation of LA and recorded at −70;mV. Scale bars: 30 ms, 40 pA. **k** No difference in PPR at LA to BA synapse. WT (*n* = 11 cells in 4 mice), KO (*n* = 9 cells in 5 mice); *p* > 0.05. **l** No difference in PPR at different ISIs. WT and KO. WT (*n* = 10), KO (*n* = 7); two-way ANOVA, F (1, 15) = 2.182, *p* > 0.05.

Although the LA is a critical site of synaptic plasticity in fear learning, BA is a central integrator of emotional context^21,36^. The BA is driven by the LA and regulated by inputs from the mPFC to provide executive control over fear behavior^27,37,38^. In particular, neurons in the dmPFC send direct glutamatergic inputs to the BA to control fear expression^20,31^. We therefore evaluated synaptic inputs from the dmPFC and LA to the BA in *NRXN1α* KO mice, using whole-cell patch-clamp recording at −70 mV in coronal slices. To investigate dmPFC–BA synapses, we infused AAV vectors expressing a ChR2-EYFP fusion protein under synapsin promoter (AAV1-hSyn-ChR2(H134R)-EYFP) into the dmPFC (Fig. 1a, b). Optogenetic activation of channelrhodopsin in dmPFC terminals in the presence of 10 µM Bicuculline showed that paired pulse ratio (PPR) of EPSCs recorded from BA principal neurons of *NRXN1α* KO was strongly reduced, particularly at short ISIs (Fig. 1d–f). The reduced PPR at dmPFC–BA synapses suggests altered transmitter release probabilities in *NRXN1α* KO mice. To test whether this translated to altered synaptic strength at dmPFC–BA pathway, we performed input–output analysis at several stimulation intensities. We found that the amplitude of AMPA EPSCs was smaller across all stimulation intensities in *NRXN1α* KO mice (Fig. 1g, i).

To assess the effect of *NRXN1α* deletion on LA–BA pathway, we stimulated LA with bipolar tungsten electrodes and found that the evoked EPSCs in BA principal neurons showed no difference in PPR between the WT and *NRXN1α* KO mice (Fig. 1j–l).

**Fig. 2:**
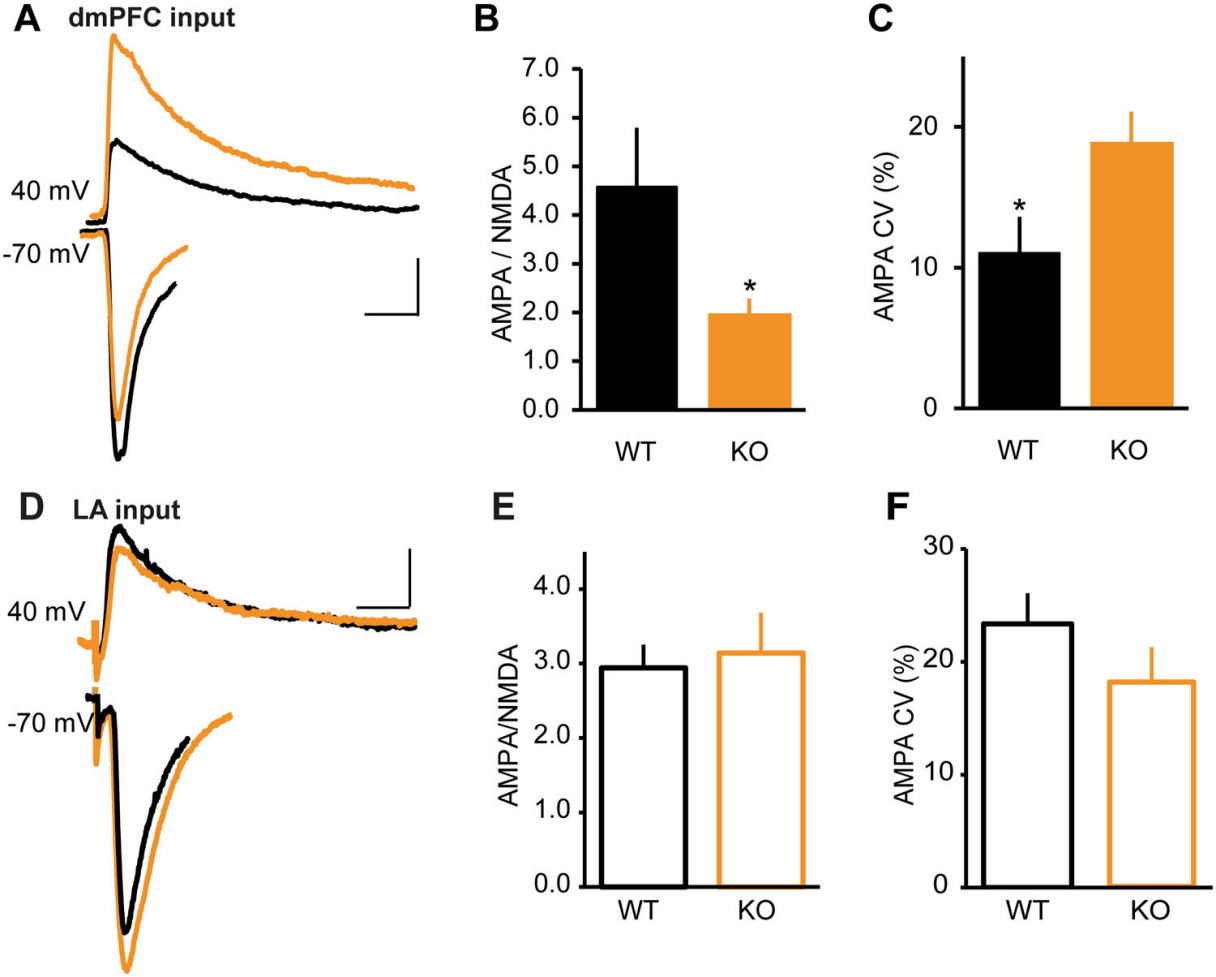
Decreased AMPA/NMDA ratio at dmPFC–BA synapse in *NRXN1α*-KO mice. **a**–**c** AMPA- and NMDA-mediated EPSCs at dmPFC–BA synapse. WT (*n* = 11 cells in 6 mice), KO (*n* = 12 cells in 6 mice). **a** Sample traces of light evoked AMPA receptor EPSCs at −70 mV and NMDA current measured at +40 mV, 40 ms after stimulation onset. Scale bars: 40 ms, 40 pA. **b** Decrease in AMPA/NMDA ratio in KO mice; *p < 0.05. **c** Stimulation intensity was adjusted to obtain comparable AMPA EPSC amplitudes in WT and KO mice. Increased AMPA EPSC coefficient of variation (CV) in KO mice; *p < 0.05. **d**–**f** AMPA- and NMDA-mediated currents at LA–BA synapse. WT (*n* = 13 cells in 6 mice), KO (*n* = 10 cells in 5 mice). **d** Sample traces of AMPA and NMDA EPSCs. Scale bars: 30 ms, 40 pA. **e** No difference in AMPA/NMDA ratio between WT and KO; *p* > 0.05. **f** No difference in AMPA EPSC CV between WT and KO; *p* > 0.05.

To search for alterations in postsynaptic function, we first adjusted stimulation intensities in WT and *NRXN1α* KO mice to obtain comparable AMPA EPSC amplitudes (Supplementary Fig. S1A, B), and measured *N*-methyl-d-aspartate (NMDA) currents at +40 mV, 40 ms after stimulation onset. We then analyzed AMPA to NMDA ratio at dmPFC to BA and at LA to BA synapses. *NRXN1α* KOs had a greater than twofold reduction in the AMPA/NMDA ratio at the dmPFC–BA pathway (Fig. 2a, b). Although this could be due to a decrease in AMPA EPSC amplitude or an increase in NMDA EPSC amplitude, several findings suggest that a decrease in AMPA EPSCs is partly responsible for this change. First, our data in Fig. 1g–i show that amplitude of AMPA EPSCs is reduced at various stimulation intensities in *NRXN1α* KO mice. Second, the coefficient of variation (CV) of AMPA EPSC amplitude was larger in *NRXN1α* KO mice, indicating ensemble sampling from a smaller group of stochastic channels (Figs. 1i and 2c). To test whether increase in NMDA EPSCs contributed to the reduced AMPA/NMDA ratio in *NRXN1α* KO mice, we compared NMDA EPSCs in WT and KO mice, and found a significant enhancement in *NRXN1α* KO mice (Supplementary Fig. S1C).

In contrast to our findings at dmPFC–BA synapses, there was no difference in AMPA/NMDA ratio, AMPA CV (Fig. 2d–f), or NMDA EPSC amplitudes (Supplementary Fig. S1D) in the LA–BA pathway of WT and KO mice.

Together, our data suggest that a decrease in AMPA current and an increase in NMDA current are responsible for the reduced AMPA/NMDA ratio observed at dmPFC–BA synapses of *NRXN1α* KO mice.

### *NRXN1α* KO elicited decreased inhibitory synaptic strength at dmPFC to BA and LA to BA synapses

**Fig. 3:**
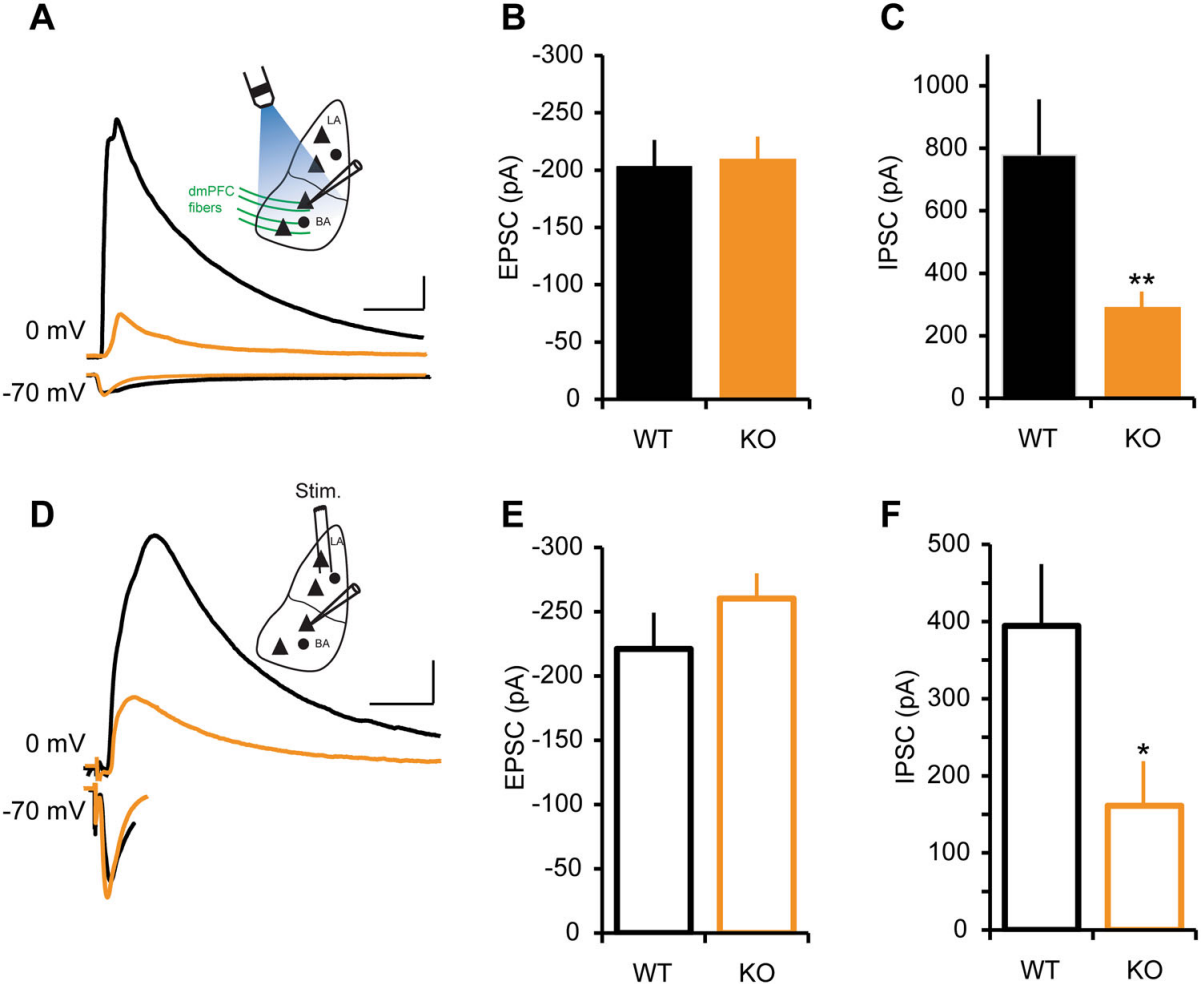
Reduced inhibitory transmission in *NRXN1α*-KO mice. **a**–**c** Inhibitory transmission in dmPFC–BA pathway. Stimulation intensity was adjusted to obtain comparable AMPA EPSC amplitudes in WT and KO mice and the consequent IPSC amplitudes were analyzed. EPSCs were recorded at −70 mV and IPSCs at 0 mV. WT (*n* = 13 cells in 7 mice), KO (*n* = 14 cells in 5 mice). **a** Representative traces of EPSCs and IPSCs from dmPFC–BA pathway in WT and KO mice. Inset: schematic diagram showing light activation of ChR2-expressing dmPFC fibers and whole-cell patch-clamp recording from a BA principal cell. Scale bars: 50 ms, 200 pA. **b** Normalize dmPFC-EPSC amplitude in WT and KO mice; *p* > 0.05. **c** Decreased IPSC amplitude in KO; **p < 0.01. **d**–**f** Inhibitory transmission in LA–BA pathway. WT (*n* = 13 cells in 5 mice), KO (*n* = 11 cells in 3 mice). **d** Representative traces of EPSCs and IPSCs recorded from LA–BA pathway in WT and KO mice. Scale bars: 50 ms, 100 pA. Inset: schematic diagram showing bipolar electrode stimulation of LA and whole-cell patch-clamp recording from a BA principal cell. **e** Normalize LA-EPSC amplitude in WT and KO mice; *p* > 0.05. **f** Mean IPSC amplitude is reduced in KO; *p < 0.05.

A tight balance between excitation and inhibition (E/I) in a synaptic network is important for normal brain function, and disturbed E/I balances have been implicated in various brain disorders^39^. To determine whether the observed deficit in excitatory transmission alters E/I balance in BA, we assessed afferent excitation-driven inhibition at dmPFC to BA (Fig. 3a–c and Supplementary Fig. S2A) and LA to BA synapses (Fig. 3d–f and Supplementary Fig. S2B). Evoked EPSCs were isolated in voltage‐clamp mode at −70 mV, which is the chloride-reversal potential of our intracellular solution, and IPSCs were recorded at 0 mV in the same neuron in drug-free ACSF. The latencies of evoked EPSCs were consistent with monosynaptic activation (Supplementary Fig. S2C-F) as previously reported^30^. In contrast to the input-specific changes in excitatory transmission onto BA principal neurons, inhibitory transmission was globally disrupted in *NRXN1α* KO mice. The amplitude of IPSCs onto BA neurons, driven either by LA or dmPFC stimulation (Fig. 3c, f), was less than half that of the controls, even when normalized by AMPA EPSC amplitude (Fig. 3b, e). These changes were also evident in the E/I ratio (Supplementary Fig. S2A, B).

### *NRXN1α* KO revealed decreased inhibitory connectivity in the BA

**Fig. 4:**
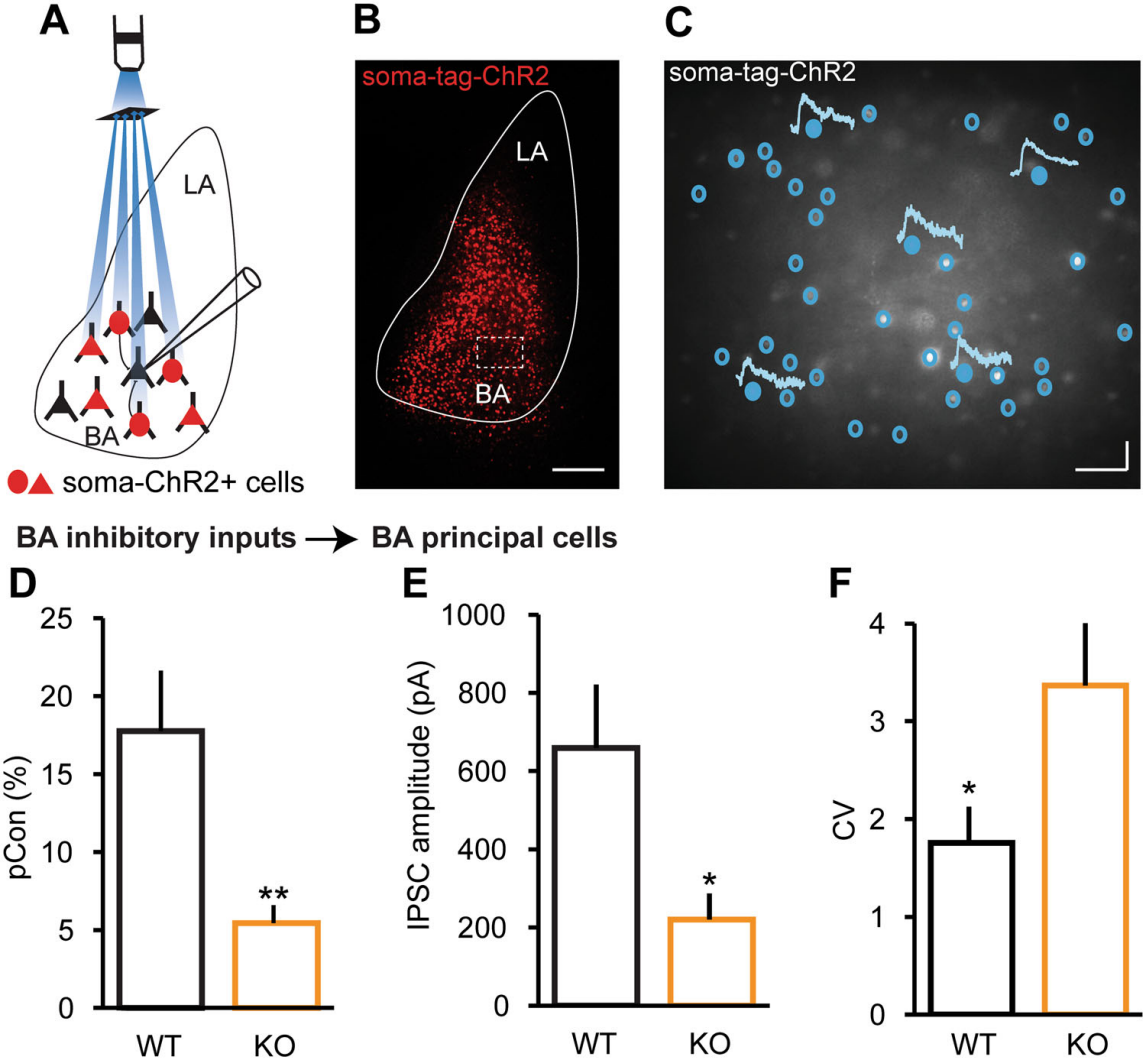
Reduced inhibitory connections in *NRXN1α*-KO mice. Local circuit mapping of inhibitory connections in the BA using digital micromirror device and soma-targeted ChR2. WT (*n* = 14 recorded cells in 6 mice), KO (*n* = 13 recorded cells in 6 mice). On average, 69 presynaptic neurons were stimulated per recorded (postsynaptic) neuron in WT mice, while 71 presynaptic neurons were stimulated per recorded (postsynaptic) neuron in KO mice. **a** Schematic diagram illustrating patterned light stimulation of soma-targeted ChR2-expressing cells while recording from a BA principal cell during circuit mapping. **b** Post-recording slice showing ChR2-expressing cells in the BA. Scale bar: 150 μm. **c** Sample traces of IPSCs recorded from BA principal neurons during circuit mapping. Filled circles represent ChR-expressing neurons that were stimulated and evoked a postsynaptic response in the recorded neuron, whereas empty circles represent ChR2-expressing neurons that were stimulated but no postsynaptic response. Scale bars: 100 ms, 50 pA. **d** Inhibitory connection probability is reduced in KO mice; **p < 0.01. **e** Mean IPSC amplitude is reduced, with concomitant increase in CV **(f)** in KO mice; *p < 0.05.

A change in the strength of inhibition could arise from a change in the excitatory drive onto inhibitory neurons, in the excitability of inhibitory neurons, or in GABAergic transmission itself. To begin to distinguish between these possibilities, we assessed the the impact of *NRXN1α* deletion on GABAergic transmission within the BA. Therefore, soma-targeted channelrhodopsin (AAV1-hSyn-ChR2-Kv2.1-P2A-H2B-mRuby2) was expressed in BA neurons. Because our virus construct could infect both glutamatergic and GABAergic neurons, we sought to minimize potential confounds by isolating inhibitory responses in the presence of excitation blockers (1 µM NBQX, 50 µM L-APV) at 0 mV (excitatory current reversal potential). We directly evaluated BA local circuit inhibitory connectivity by sequentially activating individual neurons expressing soma-targeted channelrhodopsin with a digital mirror spatial light modulator while measuring inhibitory synaptic responses in a non ChR2-expressing postsynaptic BA principal neuron (Fig. 4a–c). The probability of finding a functional inhibitory connection onto a principal neuron was greatly reduced in KO mice (Fig. 4d). Not only was the connection probability reduced, but also the quantal strength of individual inhibitory synapses in KO mice was a third of that of WTs (Fig. 4e), with a concomitant increase in CV (Fig. 4f).

Perisomatic inhibition from CB1R- and parvalbumin (PV)-expressing interneurons controls the activity of principal neurons in the amygdala^40,41^. To examine whether the observed deficit in inhibitory transmission was due to loss of perisomatic inhibitory terminals, we immunostained for potassium voltage-gated channel 2.1 (Kv2.1) to label perisomatic regions and counterstained for VGAT and CB1R to identify GABAergic puncta and CB1R puncta, respectively (Supplementary Fig. S3A-D). We analyzed the number of VGAT or VGAT/CB1R double labeled perisomatic puncta in ten randomly selected neurons per animal, and found no difference between WT and KO mice (Supplementary Fig. S3E, F). There was also no difference in the number of non-CB1R perisomatic puncta between groups (Supplementary Fig. S3G). These data suggest that the decrease in inhibitory connectivity observed in KO mice was not due to a loss of perisomatic inhibitory terminals.

### *NRXN1α* KO mice showed reduced fear expression

**Fig. 5:**
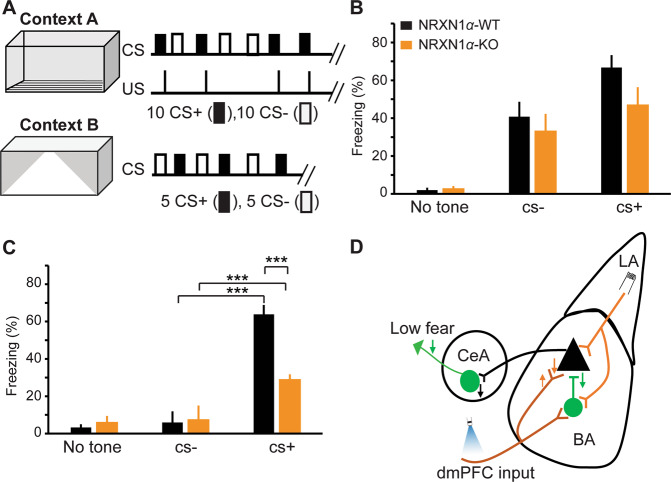
Reduced fear expression in *NRXN1α*-KO mice. **a** Schematic representation of discriminative fear conditioning paradigm. Acquisition consists of 10 tones (30 s, 50 ms pips) paired with a mild shock (CS+) and 10 interspersed tones (30 s, continuous) without a shock (CS−). Fear memory retrieval was tested in a different context one day after acquisition. Freezing was measured as a proxy for fear. **b**, **c** Steady-state freezing responses (mean of last four CSs) in WT and KO mice. **b** Freezing responses during fear acquisition. Although there was a trend towards reduced freezing during fear acquisition in KO mice, it did not attain statistical significance. WT (*n* = 9) KO (*n* = 8); two-way ANOVA, F (1, 45) = 2.995, *p* > 0.05 (0.0904). **c** Deficit in fear memory retrieval in KO mice: WT (*n* = 9), KO (*n* = 8); two-way ANOVA, F (1, 45) = 8.802, *p* < 0.01. ANOVA was performed using freezing responses to the last four CSs from each mouse. Asterisks denote significant post hoc tests. *p < 0.05, ***p < 0.001. **d** Summary and model of circuit changes in *NRXN1α*-KO mice. Although there was an increase (upward arrow) in PPR, the overall postsynaptic receptor activity was reduced at the dmPFC–BA synapses. Excitatory input from LA to BA principal cells was unaltered. Feedforward inhibition from LA or dmPFC to BA principal cells, and local inhibitory transmission within the BA were reduced (downward arrow) in *NRXN1α*-KO mice. Failure of dmPFC inputs to effectively drive fear-related BA microcircuit could result in a reduced recruitment of CEA fear microcircuit, ultimately leading to low fear. Orange and green arrows represent excitatory and inhibitory effects, respectively.

Inhibition plays a central role in gating the activity and plasticity of excitatory microcircuits that govern fear learning and memory in the amygdala^42,43^. To examine whether deletion of *NRXN1α* affects fear learning and memory, we subjected mice to discriminative fear conditioning in which two different tones were delivered to the mice in random order with one tone paired with a mild shock (CS^+^) and the other not (CS^−^) (Fig. 5a). WT mice could clearly distinguish between CS^+^ and CS^−^ during conditioning (Supplementary Fig. S4A), but freezing responses to CS^+^ and CS^−^ were not significantly different in KO mice (Supplementary Fig. S4B). However, both WT and KO mice could clearly distinguish between the tones during fear memory retrieval, as they froze selectively to the CS^+^ tone when tested 1 day later in a different context (Supplementary Fig. S4C, D). To compare the extent of CS+ evoked freezing between WT and KO mice, we analyzed the steady-state freezing responses (mean of last four CS+). Although mean freezing response to CS+ was not different between WT and KO mice during fear acquisition (Fig. 5b), *NRXN1α* KO mice had severely reduced CS^+^ evoked freezing during fear memory retrieval (Fig. 5c). These findings cannot be attributed to differences in locomotor activity, as there was no difference in average motion between WT and KO mice (Supplementary Fig. S4E). Together, our data suggest that *NRXN1α* KO mice have poor fear memory retrieval.

Taken together, the combined deficits in excitatory synaptic strength at the dmPFC–BA pathway and the abnormal inhibitory transmission within the BA likely contribute to the observed defects in fear memory retrieval (Fig. 5d). The synapse class-specific deficits in excitatory transmission onto BA principal neurons, with dmFPC inputs compromised and LA inputs largely unaffected, highlights the need to understand the role of NRXNs at the level of individual classes of synapses.

## Discussion

*NRXN1α* is a high-confidence candidate gene for both autism and schizophrenia but how mutations in *NRXN1α* alter synaptic circuits in specific brain regions contributing to the symptoms of these disorders is not known. In this study, we examined the synaptic connection from the dmPFC and LA to BA neurons and found that the excitatory synaptic strength of dmPFC to BA neurons was reduced in *NRXN1α* KO mice, as seen in the input–output function and AMPA/NMDA ratio. Synaptic transmission from LA to BA principal neurons was not affected. In addition, inhibition was compromised in the BA regardless if it was driven by dmPFC or LA inputs.

Because of differential expression of individual neurexins and the many possible postsynaptic binding partners with unique affinity for each neurexin flavor, the neurexins may have distinct functions at different classes of synapses^44^. In support for synapse class-specific roles for neurexins, we found that the excitatory synaptic connection from dmPFC to BA depends critically on the presence of *NRXN1α*, but the synapse from LA, the main upstream synaptic input, to the same BA neurons was not affected. At the dmPFC to BA synapse, knocking out *NRXN1α* had both pre and postsynaptic consequences. That two classes of synapses onto the same neuron would have a differential dependence on a given neurexin is not surprising given that neurexins are expressed presynaptically, and therefore the presynaptic neuron dictates the neurexin composition of the synapse.

The interpretation that the amplitude of AMPA mediated responses was reduced at the dmPFC to BA synapse is based on the conjunction of the following observations. First, the amplitude of AMPA responses is decreased at all stimulation intensities evaluated, which is congruent with the reduced AMPA/NMDA ratio. In addition, the CV in AMPA current amplitude is larger, indicating fewer channels are contributing to the stochastic sample. The dependence on NRXN1α for promoting synaptic AMPA receptor insertion or stabilization was initially shown in the hippocampal CA1 pyramidal neurons and in cerebellar climbing fibers synapses^45,46^.

The mechanism of *trans*-synaptic stabilization of AMPA receptors by NRXN is not well understood but the leucine-rich repeat transmembrane neuronal proteins, a postsynaptic NRXN binding partner family, directly bind to AMPA receptors^47–49^. Also, the neuroligins may stabilize them indirectly through their binding to postsynaptic scaffolding proteins, which themselves anchor AMPA receptors^50^. In addition to *NRXN1*, knocking out *NRXN3α/β* in hippocampal neurons also reduced AMPA current amplitude and was associated with increased internalization of GluA1 AMPA receptors, an effect that could be rescued by any NRXN lacking SS4 even when the c-terminal portion was replaced with a glycosylphosphatidylinositol anchor^51,52^. Placing our results in the context of these studies, it is tempting to speculate that synaptic terminals arising from dmPFC and synapsing on BA neurons predominantly express NRXN1α and that SS4− is the major splice variant.

We also found increased NMDA currents at the dmPFC–BA synapse of *NRXN1α* mice. *NRXN1α* deletion could exert a trans-synaptic effect on NMDA-receptor activity in the postsynaptic neuron or via a cell-autonomous postsynaptic effect. Although the mechanism behind NMDA current enhancement was not investigated in this study, previous study in neocortical slices of triple α-neurexin KO mice reported a depressed postsynaptic NMDA-receptor function mediated by a cell-autonomous mechanism^53^.

We found a strong decrease in PPR at the dmPFC–BA synapses in *NRXN1α* KO mice, suggestive of altered vesicle release probability. The molecular mechanism for the altered presynaptic release properties is also not clear. Given that NRXNs interact with key regulators of vesicular release such as CASK^54^, Mint^55^, and presynaptic N- and P/Q-Type calcium channels^56^, there are many potential sites of release modulation. Knocking out all *α-NRXNs* severely reduces the spontaneous, action potential evoked release of both glutamate, and GABA^12^. Furthermore, short-term depression of release is exacerbated at short ISIs in triple *NRXN* KOs^12^. These deficits are attributed to decreased N-Type calcium channel mediated influx at the synaptic terminal^12^. The critical domain for the regulation of presynaptic N-Type calcium channels is in the N-terminal domain of the α-neurexins given that *NRXN1α* but not NRXN1β rescues N-type and P-type calcium currents mediating synaptic responses in brain stem^56^. The reduction in PPR we demonstrate at the dmPFC to BA synapse in the *NRXN1α* KO was not observed at the LA to BA synapse or in CA1 pyramidal neurons, of the *NRXN1α* KO^45^. Likewise, in conditional *NRXN3α/β* KO mice PPR was not affected at excitatory synapses from CA1 neurons onto burst firing or regular firing subiculum pyramidal neurons, or at excitatory synapses onto olfactory bulb neurons^51,52^. However, the precedence for single neurexin isoform determination of release properties has been previously reported. Both *NRXN2α* and *NRXN2α/β* KO mice had decreased mEPSC frequency in layer V pyramidal neurons in somatosensory cortex and reduced PPR^57^. Again, these results underscore the synapse class-specific role of *NRXNs*. Surprisingly, the reduced PPR we found in *NRXN1α* KO mice did not translate to increased postsynaptic activity, rather a decrease in postsynaptic function. Although we report differences in the effect of *NRXN1α* KO on optogenetically recruited dmPFC input and electrically stimulated LA input to BA neurons, we cannot exclude potential confounds due to differences in stimulation methods. It is however interesting to note that despite the differences in stimulation methods, the unique effect of *NRXN1α* KO on dmPFC inputs to BA neurons was only observed on the direct excitatory input and not on the associated feedforward inhibition. Future studies using optogenetic stimulation to recruit multiple synaptic inputs onto the same neuron will ultimately resolve the putative synapse class-specific regulation of excitatory synapses by a NRXN.

We found that inhibition in BA was severely compromised in the *NRXN1α* KOs regardless of whether the inhibition was driven from dmPFC or LA afferents. Our data suggest that this reduction in the strength of inhibitory transmission is due in part to a decrease in the probability of connection from local inhibitory neurons to BA principal neurons and in part to a reduction in the strength of this GABAergic connection. It could also be reduced excitatory drive to individual inhibitory neurons as well as reduced intrinsic excitability, but these were not evaluated in this study. Our immunohistological experiments showed that the decrease in inhibitory transmission in the BA of *NRXN1α* KO mice was not due to a decline in the number of perisomatic inhibitory synapses, as there was no difference between WT and KO groups. In contrast, triple KO of all three α-neurexins resulted in a reduction in the density of VGAT puncta and symmetrical synapses^12^. However, this could not account for the larger decrease in synaptic transmission in *NRXNα* KO mice, rather it was attributed to impaired calcium channel function, especially the N-type. In terms of postsynaptic regulation, α-neurexins expressed in heterologous cells co-cultured with hippocampal neurons induce clustering of gephrin, neuroligin 2 and GABAR γ2^58^. In addition, neurexins directly interact with postsynaptic GABAA α 1 receptors, further highlighting their potential to regulate inhibitory synapse function^59^.

The question then arises as to what type of presynaptic inhibitory neurons governs the regulation of inhibitory synapses by neurexins. Conditional deletion of both the α- and β-isoforms of all three neurexins in PV neurons had quite different effects on the inhibitory synapse from PV neurons to layer V pyramidal neurons in mPFC than did the same triple KO in somatostatin (SOM) neurons at the synapse from the SOM neurons to the same population of mPFC Layer V neurons^14^. In the case of the PV KO neurons, there was a dramatic loss of synapses, and a reduction in the amplitude of unitary evoked inhibitory current but no presynaptic consequences on spared synapses. On the other hand, SOM KO had no reduction in synapse number, but had reduced probability of release and reduced presynaptic terminal calcium influx per action potential in addition to a postsynaptic decrease in unitary IPSC amplitude. Although, we do not know the identity of the BA inhibitory neurons affected by *NRXN1α* KO, a reduction in the number of terminals from perisomatic inhibitory neurons can be excluded, based on our findings.

### Circuit consequences on fear learning and memory

In terms of behavior, *NRXN1α* KO mice displayed impaired fear memory retrieval 24 h after discriminative fear conditioning. This is in contrast to what was found in *NRXN1α* KO rats^60^. *NRXN1α* KO rats had normal fear memory retrieval 48 h after classical fear conditioning but impaired social fear learning. The dissimilarity in experimental protocols, and specie-related differences might account for the disparity. Locomotor activity is a potential source of confounds when using freezing behavior as proxy for fear, whereas Grayton et al., 2013, reported reduced locomotor activity in *NRXN1α* KO mice^61^, another study detected no significant locomotor phenotype^45^. We found no difference in average motion between WT and *NRXN1α* KO mice. Furthermore, reduced locomotor activity would result in increased freezing, not reduced freezing as observed in the present study. Another source of confound when using freezing as a measure for fear behavior is the presence of active defensive behaviors such as jumping and escape behaviors. The shock intensity used in this study did not induce any active defensive behavior and we did not observe such during fear acquisition or memory retrieval. Behavioral changes upon fear conditioning are thought to rely on synaptic plasticity at sensory afferents onto LA principal neurons, causing enhanced neuronal responses to the auditory CS^43,62^. During fear conditioning, BLA (LA and BA) PV interneurons are excited by the auditory CS and inhibit SOM interneurons leading to a dendritic disinhibition of BLA PNs and enables dendritic processing of the CS^43,63^. Recently, it was reported that vasoactive-intestinal peptide-positive interneurons provide adaptive disinhibitory gating during associative learning^64^. Although there was a trend towards reduced freezing during fear conditioning in *NRNX1α* KO mice, it however did not attain statistical significance; rather a more robust reduction in freezing was observed during fear memory retrieval. Our study focuses on the functional consequence of *NRNX1α* deletion in the BA, a future study examining the effect of NRNX1α deletion on specific interneuronal subtypes and their interactions within the LA and BA will shed more light on the overall impact of *NRNX1α* deletion on amygdala circuit of fear. Furthermore, the *NRXN1α* KO is a global KO and while cued fear conditioning is mediated by amygdala circuits, fear behavior is regulated by other brain regions and the state of the animal. Therefore, it is possible that alterations due to *NRXN1α* deficiency in other brain regions contribute to the fear phenotype of these mice.

The mPFC clearly plays a central role in learned fear and its extinction, with part of dmPFC, the PL being implicated in fear expression and the infralimbic region (part of vmPFC) in fear extinction^31,65^. During fear expression, CS directly drives neurons in the PL that send excitatory projections to the BA and reciprocal connections between these regions regulate fear expression^22^. Here, we report a suppressed excitatory input from dmPFC to BA principal neurons and a dysfunctional inhibitory transmission within the BA microcircuit of *NRNX1α* KO mice. This could lead to a reduced recruitment of fear microcircuit within the CEA, thereby suppressing the fear output (Fig. 5d).

In summary, our study shows that the synapse from the dmPFC to the BA, which regulates fear expression, is dependent on the autism and schizophrenia susceptibility gene, *NRNX1α*. Furthermore, inhibition in the BA, a central integrating structure for fear and anxiety, is decimated by the loss of *NRNX1α.*

## Supplementary information

Supplementary Figure legends

Figure S1

Figure S2

Figure S3

Figure S4
