# Supplementary material for: Deletion of NRXN1α impairs long-range and local connectivity in amygdala fear circuit
Source: Transl Psychiatry. 2020 Jul 19;10:242. doi: 10.1038/s41398-020-00926-y (PMC7370229; doi:10.1038/s41398-020-00926-y)
Supplement: Supplementary file 1 — Supplementary Figure legends [file 41398_2020_926_MOESM1_ESM.docx]

**Supplementary Figure legends**

**Fig. S1. Properties of EPSCs from dmPFC-BA and LA-BA synapses.** Stimulation intensity was adjusted to obtain comparable AMPA EPSC amplitudes in WT and KO mice. **(A)** Normalized mean AMPA receptor–mediated EPSC amplitudes of dmPFC–BA inputs, associated with figure 2B and 2C. WT (n = 11 cells in 6 mice), KO (n = 12 cells in 6 mice); p > 0.05. **(B)** Normalized mean AMPA EPSC amplitudes of LA–BA input, associated with figure 2E and 2F. WT (n = 13 cells in 6 mice), KO (n = 10 cells in 5 mice); p > 0.05. **(C)** Increased NMDA currents at dmPFC-BA synapse of KO mice at stimulus intensity required to normalize AMPA current amplitude in **‘A’.** **p < 0.01. **(D)** No difference in NMDA current amplitude at LA-BA synapse of KO mice at stimulus intensity required to normalize AMPA current amplitude in **‘B’**; p > 0.05.

**Fig. S2.** **Excitation–inhibition (E/I) ratio and synaptic latency in WT and NRXN1α KO mice.** **(A)** There was a trend towards increase in E/I at dmPFC–BA pathway, which is consistent with the reduced IPSC amplitude in KO mice (figure 3C) ; WT (n = 13 cells in 7 mice), KO (n = 14 cells in 5 mice), p = 0.09. **(B)** E/I was significantly increased at LA–BA pathway, in line with figure 3F; WT (n = 13 cells in 5 mice), KO (n = 11 cells in 3 mice), p < 0.05. **(C and D)** No difference in latency and corresponding synaptic jitter of dmPFC input to BA between WT and NRXN1α KO mice. WT (n = 13 cells in 7 mice), KO (n = 14 cells in 5 mice), p>0.05. **(E and F)** No difference in latency and corresponding synaptic jitter of LA input to BA between WT and NRXN1α KO mice. WT (n = 13 cells in 5 mice), KO (n = 11 cells in 3 mice), p>0.05.

**Fig. S3: The number of GABAergic perisomatic puncta is unaltered in NRXN1α KO mice** (**A-D)**. Top: Visualization of GABAergic perisomatic puncta in the BLA of a WT mouse in a single plane confocal image. Immunostaining against: **(A)** Kv2.1 to delineate perisomatic regions; **(B)** VGAT for GABAergic terminals; and **(C)** CB1R. **(D)**. Overlay of ‘A-C’. Analyzed neurons are highlighted in circle**. (A-D)**. Bottom: Similar to ‘A-D’ but in a KO mouse. **E-G.** Quantification of perisomatic puncta. WT (n = 6 mice), KO (n = 5 mice). 10 cells per animal. No difference in the number of **(E)** VGAT puncta; **(F)** CB1R puncta; **(G)** non-CB1R puncta. Scale bar: 40μm.

**Fig. S4. Discriminative fear conditioning in NRXN1α WT and KO mice.** **(A)** Significant difference in freezing to CS+ and CS- during fear acquisition in WT mice (n = 9, two-way ANOVA, F (1, 16) = 8.377, p < 0.05). **(B)** No difference in freezing to CS+ and CS- during fear acquisition in KO mice (n = 8). Two-way ANOVA, F (1, 14) = 0.9811, p > 0.05. **(C)** Significant difference in freezing to CS+ and CS- during fear memory retrieval in WT mice; n = 9, two-way ANOVA, F (1, 16) = 122.7, p < 0.001. **(D)** Significant difference in freezing to CS+ and CS- during fear retrieval in KO mice. Two-way ANOVA, F (1, 14) = 6.053, p < 0.05. ANOVA was performed using freezing responses to the all CS presentations from each mouse. **(E)** Motion index as an estimate of locomotor activity in WT and NRXN1α KO mice. Average motion index during a 10-minute habituation phase. WT (n = 9 mice), KO (n = 8 mice), p > 0.05.
