# Supplementary figures and images for: Deletion of NRXN1α impairs long-range and local connectivity in amygdala fear circuit
Source: Transl Psychiatry. 2020 Jul 19;10:242. doi: 10.1038/s41398-020-00926-y (PMC7370229; doi:10.1038/s41398-020-00926-y)

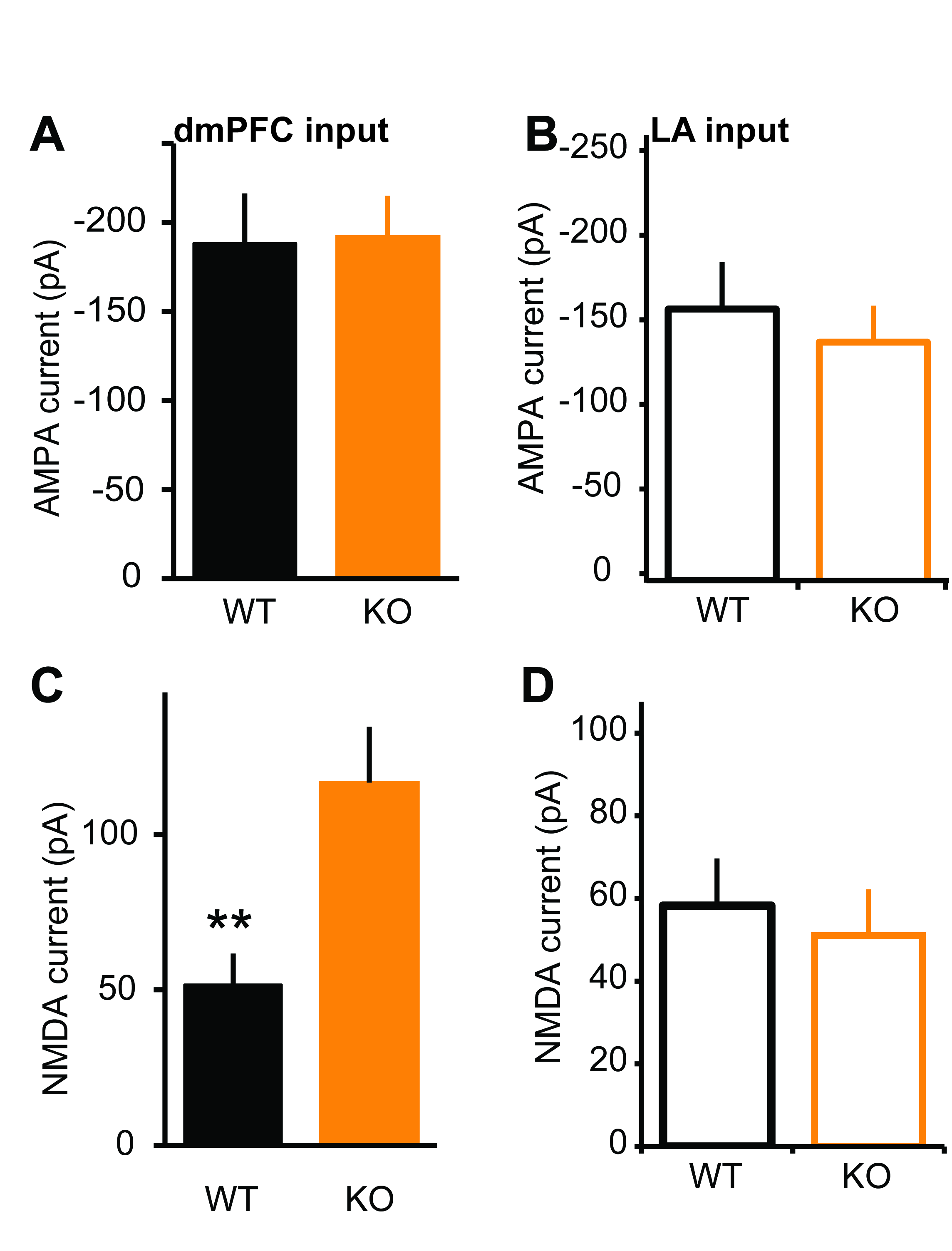

Supplement: Supplementary file 2 — Figure S1 [file 41398_2020_926_MOESM2_ESM.tif]

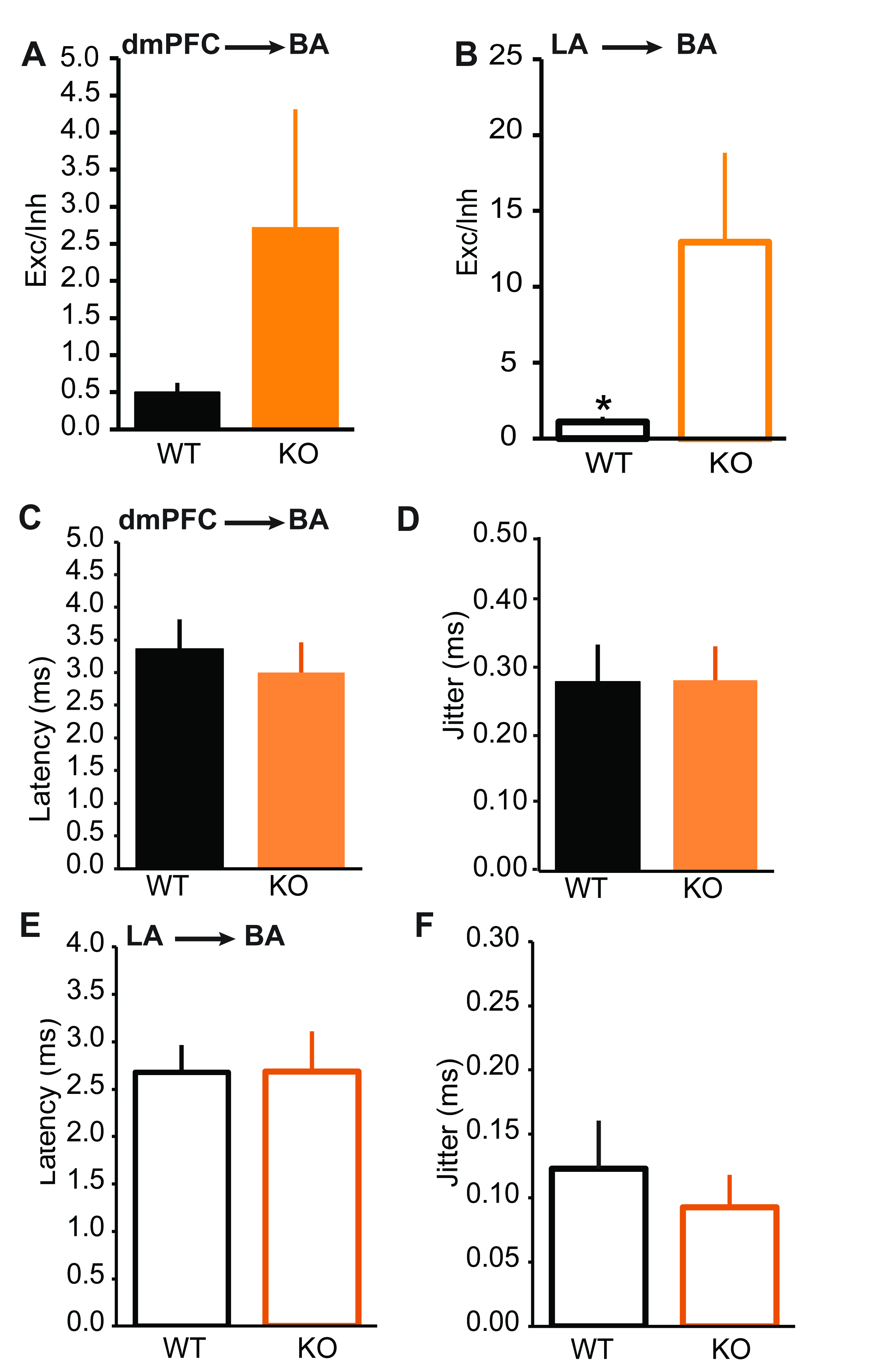

Supplement: Supplementary file 3 — Figure S2 [file 41398_2020_926_MOESM3_ESM.tif]

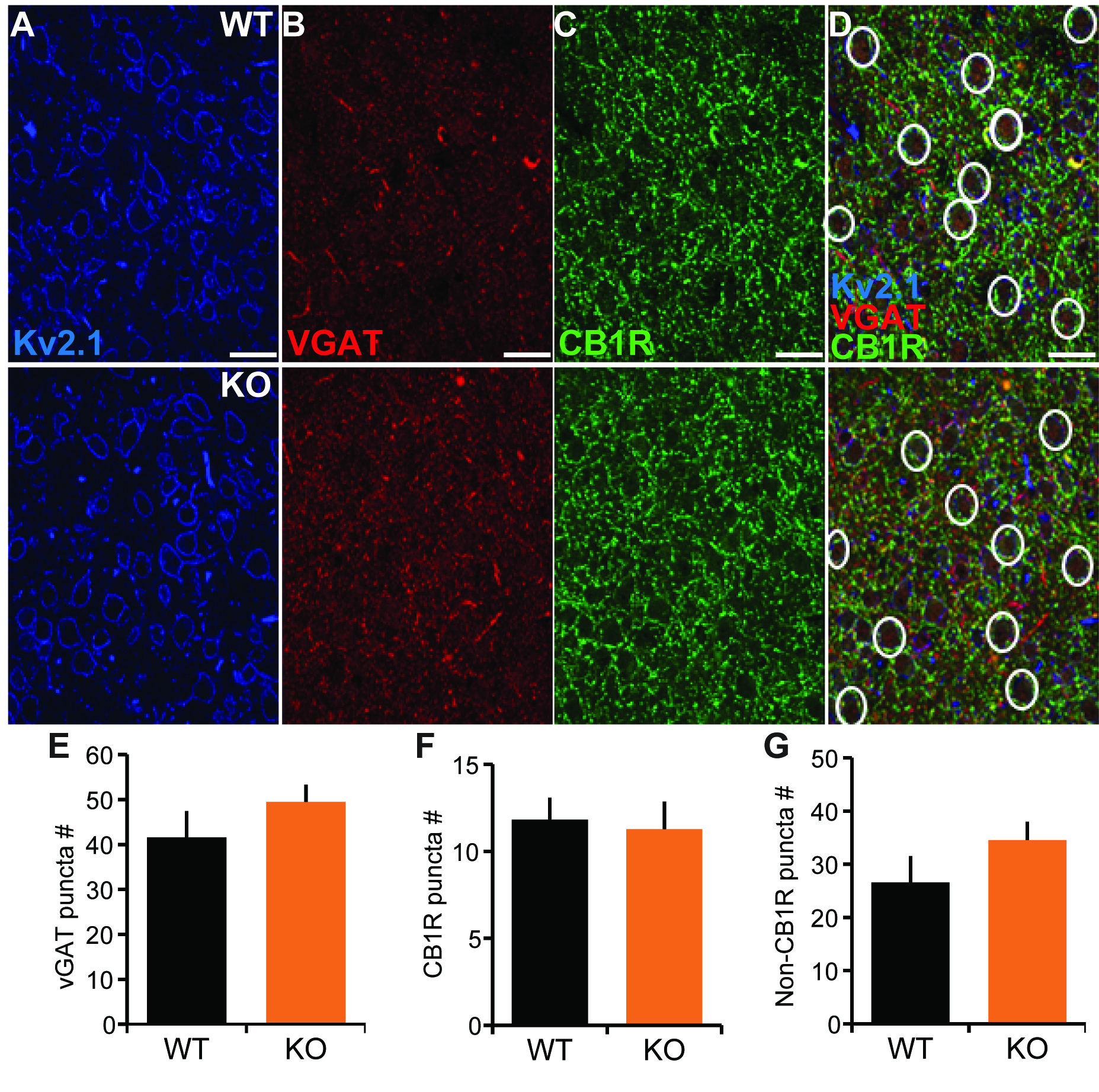

Supplement: Supplementary file 4 — Figure S3 [file 41398_2020_926_MOESM4_ESM.tif]
